# Circular of the American Medical Society in Paris

**Published:** 1852-03

**Authors:** 


CIRCULAR.

*Paris, France, January* 10*th*, 1852.

At a recent meeting of the American physicians in Paris, an association was established, whose object is the promotion of medical science. This association, essentially national, is now progressing under the most favorable auspices; it is intended to be permanent in its nature, and is designated the "*American Medical Society in Paris.*"

Notwithstanding the vast advantages afforded by the French metropolis for the study of medical and surgical science, we feel ourselves isolated from our national medical literature, and, therefore, confidently appeal to the conductors of American journals and periodicals.

We do this with the less hesitation, feeling assured, that it will be not only a medium of improvement to ourselves, but a means of a more general diffusion and just appreciation of American literature.

By order of the Society,

ALEXANDER J. SEMMES, M. D., of D. C.
*Corresponding Secretary of the American Medical Society in Paris.*

Secular and professional journals in the United States, are very respectfully requested to copy the above circular.

P. S. In accordance with the resolutions of the American Medical Society in Paris, Dr. R. M. Jones, of Lexington, Ky., and Dr. Alexander J. Semmes, of Georgetown, D. C., have been appointed two of three delegates authorized. These gentlemen will sail from Liverpool in time to present their credentials and request seats in the Convention.

